# 

**Published:** 1853-05

**Authors:** 


### CONTENTS OF THE MAY NUMBER.

### ORIGINAL COMMUNICATIONS.

Page.

Art. I.—On the Use of Tobacco in producing Sciatica. By Harvey Jewett, M. D., Canandaigua, N. Y., - - - - 721
Art. II.—A case of Poisoning by Arsenious Acid. Reported by J. Bryant Smith, M. D., of Louisville, Ky., - - - - 730
Arr. III.—Extensive Fracture of the Cranium, with Extravasation of Blood. Death occurring in fifteen hours. Read before the Buffalo Medical Association. By James M. Newman, M. D., - - - - 733
Art. IV.—Necrosis of Femur resulting in a malignant degeneration of the soft parts requiring amputation. By C. D. Robinson, M. D., of Almond, Allegany county, N. Y., - - - 736
Art. V.—Case of Ununited Fracture, cured by Subcutaneous Perforation. By Harvey Jewett, M. D., Canandaigua, N. Y., - - - - 738
Art. VI.—Hats and Baldness. By Subscriber, - - - - 739

### ECLECTIC DEPARTMENT.

Notes taken from Lectures of M. Trousseau, on Croup and Diphtherite, - - - - 740
Dislocation of the Os Humeri, upon the Dorsum Scapulæ, - - - - 747
Treatment of Epilepsy, - - - - 749
Employment of the Iodide of Potassium as a remedy for the Affections by Lead and Mercury, - 750
Arsenical Confectionery, - - - - 754
Cholera, - - - - 755
Treatment of Dysentery, - - - - 755
Treatment of Sprains by "Firing," - - - - 756

### EDITORIAL DEPARTMENT.

The Transactions of the American Medical Association, for 1852, - - - - 757
Obituary Notice of the late William Power, M. D., of Baltimore, - - - - 760
Death of Dr. Graves, - - - - 763
Introductory and other Addresses, - - - - 765
Foundation of a Museum and Prizes, by Orfila, - - - - 767
Dr. Bennet Dowler, - - - - 768
The Obstetric Catechism, - - - - 769
Spiritual Rappers, - - - - 769
The American Psychological Journal, - - - - 770
The Lights and Shadows of Medical Life, - - - - 770
Medical College of Buffalo, - - - - 771
Prof. Hamilton's new views on Provisional Callus, - - - - 772
Proceedings of the Medical Association of the State of Alabama, - - - - 773
A Clinical Phrasebook in English and German, - - - - 774
The Virginia Med. and Surg. Journal, - - - - 774
Anatomical Bill, - - - - 775

